# Effect of *Funneliformis mosseae* and Cu Additives on the *Astragalus sinicus* Root Growth and Cd Uptake Under the Modeled Conditions

**DOI:** 10.3390/microorganisms13051109

**Published:** 2025-05-12

**Authors:** Yuxin Li, Rui Cai, Jindian Hu, Hongling Liu, Xiancan Zhu

**Affiliations:** 1 Anhui Provincial Key Laboratory of Molecular Enzymology and Mechanism of Major Metabolic Diseases, College of Life Sciences, Anhui Normal University, Wuhu 241000, China; liyuxin053@ahnu.edu.cn (Y.L.); 2121021671@ahnu.edu.cn (R.C.); 133555585348@163.com (J.H.); 2 Anlu Forestry Bureau, Anlu 432600, China; 3 Sichuan Provincial Key Laboratory of Development and Utilization of Characteristic Horticultural Biological Resources, College of Chemistry and Life Sciences, Chengdu Normal University, Chengdu 611130, China

**Keywords:** arbuscular mycorrhizal fungi, Cd stress, Cd translocation and bioconcentration, heavy metal pollution, osmotic regulation

## Abstract

Cadmium (Cd) contamination in soil poses a serious threat to plant growth and productivity, while arbuscular mycorrhizal (AM) fungi play a vital role in enhancing plant growth, improving tolerance to heavy metals, and restoring polluted ecosystems. To enhance the tolerance of *Astragalus sinicus* to Cd stress, a pot experiment was conducted to investigate the effects of inoculation and copper (Cu) addition on growth, Cd accumulation, and translocation under Cd-stressed soil conditions. The results showed that Cd inhibited the root growth of *A. sinicus*, and AM fungi inoculation and Cu + AM significantly increased root biomass and root volume (*p* < 0.05). Under Cd stress, AM fungi inoculation reduced Cd concentration by 72.40% in the shoots, while it increased by 92.69% in the roots. Both AM fungi inoculation and Cu + AM enhanced Cd uptake in the roots, while inhibiting Cd translocation to the shoots. After the application of Cu and inoculation with AM fungi, the roots have a strong absorption and enrichment ability for Cd; the bioconcentration factor of Cd in the roots of *A. sinicus* reached 1018.59% and 366.08%, respectively. Cu + AM increased the enrichment of Cd in the roots and restricted its translocation to the shoots. Moreover, the combination of AM fungi inoculation and Cu addition significantly increased soluble sugar (by 77.29%) and proline contents (by 445.62%) and reduced CAT activity (by 74.67%) under Cd stress. In summary, both Cu addition and AM fungi inoculation promoted the growth of *A. sinicus* under Cd stress, improved its physiological metabolism, and reduced Cd content in the soil, with the combined Cu and AM fungi treatment showing the most significant effect.

## 1. Introduction

With the discharge of industrial waste, fuel combustion, sewage irrigation, and improper application of chemical fertilizers and pesticides, a significant portion of agricultural land has been contaminated. Soil is vital for food supply; toxic metal pollution like that of As, Cd, Cu, Ni, and Co is often overlooked [1,2]. It reduces yields and poses food safety risks. Despite decades of study, global data are scarce. Surveys show 19% of China’s [3] soils have such issues. Of these, the exceedance rate in China of Cd was as high as 7.0%, and the combined pollution ratio in farmlands reached 22.1% [3,4]. Cd, a common heavy metal and a non-essential micronutrient in biological metabolic processes, not only affects plant growth and development but also accumulates to toxic levels in soil due to its resistance to degradation. Eventually, it bioaccumulates in the food chain, posing serious threats to human health and ecological safety. Therefore, seeking efficient, economical, and sustainable technologies for soil Cd contamination remediation has become a research priority [5].

Arbuscular mycorrhizal (AM) fungi are soil microorganisms that form symbiotic relationships with plant roots and are widely distributed across various ecosystems [6]. AM fungi can activate oil mineral elements, promoting nutrient uptake, especially of phosphorus [7], and enhancing plant tolerance to environmental stresses, including drought [8,9], salinity [10], and heavy metal contamination [11], yet the underlying mechanisms remain incompletely understood. AM fungi help plants alleviate heavy metal toxicity and improve growth performance through various mechanisms, such as altering the chemical forms of heavy metals, enhancing the antioxidant system, and improving root structure. Studies have shown that AM fungi can significantly alleviate the toxic effects of Cd on plants [12,13,14]. By improving root morphology, enhancing nutrient uptake, and regulating the expression of stress-related genes, AM fungi help reduce the accumulation of Cd in plant tissues, thereby protecting plants from oxidative damage. Additionally, AM fungi can induce the synthesis of antioxidants [15], such as superoxide dismutase (SOD), catalase (CAT), and glutathione peroxidase (GPX), which play a key role in detoxifying reactive oxygen species (ROS) generated under Cd stress. Recent studies have also found that AM fungi strains are effective in promoting the aggregation of metal-resistant microbial communities [16]. AM fungi can enhance plant tolerance to heavy metal stress through multiple mechanisms, such as promoting the absorption of mineral elements, stabilizing the chemical forms of heavy metals in soil, improving plant antioxidant capacity, chelating heavy metals to reduce their bioavailability, and stabilizing ecosystems [17,18]. Under Cd-contaminated conditions, AM fungi not only alleviate Cd toxicity to plants but also enhance plant biomass and antioxidant performance [6,19,20,21], suggesting that AM fungi inoculation may improve phytoremediation strategies in Cd-contaminated soils.

Copper (Cu) is an essential trace element for plants, participating in various physiological processes, including photosynthesis, cell wall metabolism, ROS scavenging, and the regulation of antioxidant enzyme activity [22,23]. Appropriate levels of Cu can enhance plant resistance to stress; however, excessive Cu may induce oxidative stress, negatively affecting plant growth [24,25]. Under Cd stress, the role of Cu in plants is complex. It may alleviate Cd toxicity by activating the antioxidant system, but it could also exacerbate Cd toxicity through metal ion competition, disrupting the normal functions of certain ions in cellular metabolism. Among AM fungi species, *Funneliformis mosseae* has been extensively studied for its potential in alleviating Cu stress in plants. For instance, research by Adrees et al. (2015) [26] demonstrated that inoculation with *F. mosseae* significantly enhanced plant tolerance to Cu stress. This was likely achieved through modulating Cu uptake and translocation. *F. mosseae* seemed to restrict excessive Cu entry into plant roots and limit its transport to the shoots, thus reducing Cu induced oxidative damage to the cell membranes. However, the effectiveness of *F. mosseae* in alleviating Cu stress can vary depending on multiple factors, such as the plant species it colonizes, soil conditions, and the concentration of Cu in the environment [27,28,29]. Given this variability, exploring the interaction between Cu and *F. mosseae* under Cd stress is of great significance. Understanding these complex interactions can provide valuable insights for developing effective strategies to remediate soils contaminated with Cd and to promote plant growth in such challenging environments.

*Astragalus sinicus* L., also known as Chinese milk vetch or red clover, is an annual herbaceous legume traditionally used as a green manure crop in Chinese agriculture. It is highly stress-tolerant, widely adaptable, and capable of forming symbiotic relationships with AM fungi [30,31,32]. As an efficient phytoremediation material, *A. sinicus* shows great potential in the remediation of Cd-contaminated soils. The selection of *F. mosseae* in this study is mainly due to its wide distribution, ease of acquisition, cultivation, and ability to form good symbiotic relationships with numerous plants, posing minimal potential risks to *A. sinicus* and ensuring the stable implementation of the experiment and the reliability of the results [33,34]. Therefore, we hypothesize that inoculating *F. mosseae* and applying Cu can mitigate Cd accumulation in *A. sinicus* and promote plant growth under Cd stress. To test this hypothesis, we examined the effects of *F. mosseae* inoculation on root growth, physiological and biochemical responses, and Cd accumulation and translocation characteristics in *A. sinicus* under Cd stress conditions, providing a theoretical basis for the coremediation of heavy metal pollution by plants and AM fungi.

## 2. Materials and Methods

### 2.1. Experimental Materials and Design

Seeds of *A. sinicus* (purchased from Wuhu Nanling County Hongbao Seed Industry Co., Ltd., Wuhu, China) were surface-sterilized with 70% alcohol for 0.5 min, rinsed two times with sterile water, then disinfect with 2% sodium hypochlorite solution for 20 min, rinsed two times with sterile water, and germinated on wet filter paper in Petri dishes at 25 °C. The 3-day-old seedlings were transferred into plastic pots (10 cm in depth and 14 cm in mouth diameter) containing 0.625 kg of autoclaved (121 °C, 0.11 MPa, 2 h) growing mixture of campus soil, nutrient soil, and vermiculite (1:1:1, *v*/*v*/*v*), mixed well, and sieved through a 2 mm sieve. The potted substrate had been inoculated with *Funneliformis mosseae* before being transplanted by placing 20 g of inoculum 5 cm below the surface of the substrate. *F. mosseae* strain (soil, 176 spores/10 g, hyphae, and infected clover root fragments) was provided by the Germplasm Resource Bank of Arbuscular Mycorrhizal Fungi at the Institute of Root Biology, Yangtze University. The strain exhibits good plant growth performance under Cd-stressed conditions [35]. The soil had a pH of 5.98, organic matter content of 5.21 g/kg, total nitrogen content of 937.04 mg/kg, total phosphorus content of 588.01 g/kg, Cd content of 0.56 mg/kg, and Cu content of 52.44 mg/kg.

A greenhouse pot experiment was conducted to assess the effects of *F. mosseae* and Cu on plant growth under Cd stress. The experiment included inoculated or non-inoculated with AM fungi, application or non-application of Cu and Cd, resulting in a total of 8 treatment groups. Each treatment was replicated 8 times, with one pot per replicate, arranged in a completely randomized design, leading to a total of 64 pots. CdSO_4_ and CuSO_4_ solutions were applied at concentrations of 20 mg/kg to sterilized soil. The pots were placed in a greenhouse and cultivated for 3 months; the greenhouse was set at 25 °C, with air humidity at 40–60%, and soil moisture controlled at 20–25%. The plants were grown under a 12 h light–dark cycle. During the cultivation period, Hoagland nutrient solution was applied once or twice weekly. Checking the soil moisture every 2–3 days, when the soil moisture dropped below 20%, an appropriate amount of water was added to each pot to restore the moisture level.

### 2.2. Growth Parameters Measurement

After 90 days of Cd exposure, plants were carefully uprooted. Roots were initially rinsed under running tap water to remove bulk soil, followed by three sequential washes in ice-cold (4 °C) sterile deionized water with gentle agitation to eliminate rhizosphere particles. The root length from the surface of the substrate to the tip of the longest root was measured using a vernier caliper. The root volume was measured by the water displacement method in a graduated cylinder. The root samples were placed in an oven at 105 °C for 30 min, and subsequently dried at 80 °C until a constant weight was reached. The dry weight was measured, and the biomass was calculated. Four replicates were used per treatment. A 1 cm-long root segment was excised from the mid-portion of the root and subsequently washed with 10% KOH, and then stained in a 0.05% trypan blue solution after being placed in a water bath at 90 °C for 30 min. Microscopic examination was carried out, and the AM fungi colonization rate was calculated. Four replicates were used per treatment.

### 2.3. Heavy Metal Cd Content Measurement

Soil samples were digested using an HCl-HNO_3_-HClO_4_-HF acid digestion system, and plant samples were digested using a nitric acid–perchloric acid method. The Cd concentration was quantified by inductively coupled plasma optical emission spectrometry (ICP-OES, ICPS-7500, Shimadzu, Kyoto, Japan).

The Cd translocation factor (TF) was calculated as follows:

TF = Cd content in shoots/Cd content in roots

The Cd bioconcentration factor (BCF) was calculated as follows:

BCF = Total Cd content in root/Cd content in soil

### 2.4. Osmotic Regulator Measurement

For each treatment, four replicates were used for root measurements. The contents of soluble sugars, soluble proteins, and proline were determined using spectrophotometric methods.

Soluble sugar content: A 0.1 g of fresh root sample was extracted with 1 mL of 80% ethanol at 80 °C for 30 min in a water bath, followed by centrifugation. This extraction was repeated twice, and all supernatants were combined and adjusted to a final volume (e.g., 10 mL). A 0.5 mL aliquot of the extract was mixed with 1.5 mL of anthrone reagent, heated in a boiling water bath for 10 min, and then rapidly cooled. The absorbance was measured at 620 nm using a spectrophotometer (UV2450, Shimadzu, Kyoto, Japan), and the soluble sugar content was calculated using a standard curve [36].

Soluble protein content: A 0.1 g of fresh root sample was homogenized in 1 mL of 50 mmol/L phosphate buffer (pH 7.0) on ice. The homogenate was centrifuged at 12,000 rpm for 20 min at 4 °C, and the supernatant was collected as the protein extract. The reaction mixture (2 mL) contained 0.1 mL of extract and 1.9 mL of Coomassie Brilliant Blue G-250 reagent. Absorbance was measured at 595 nm, and soluble protein content was calculated using a bovine serum albumin (BSA) standard curve [37].

Proline content: A 0.1 g of fresh root sample was homogenized in 1 mL of 3% sulfosalicylic acid solution on ice. The homogenate was centrifuged at 12,000 rpm for 20 min at 4 °C, and the supernatant was collected. The reaction mixture (2 mL) contained 1 mL of extract, 1 mL of 2.5% ninhydrin solution, and 1 mL of glacial acetic acid. After mixing, the sample was heated in a boiling water bath for 30 min, rapidly cooled in an ice bath, and the absorbance was measured at 520 nm. Proline content was calculated using a standard curve [36].

### 2.5. Malondialdehyde (MDA) and Hydrogen Peroxide (H_2_O_2_) Contents Measurement

MDA content: A 0.1 g of fresh root sample was homogenized in 1 mL of 10% trichloroacetic acid (TCA) solution on ice. The mixture was centrifuged at 12,000 rpm for 15 min at 4 °C, and the supernatant was collected. The reaction mixture (2 mL) contained 1 mL of extract and 1 mL of 0.6% thiobarbituric acid (TBA) in 10% TCA. After mixing, the sample was heated in a boiling water bath for 10 min, rapidly cooled in an ice bath, and centrifuged. Absorbance was measured at 532 nm and 600 nm [36].

H_2_O_2_ content: A 0.1 g of fresh root sample was homogenized in 1 mL of precooled 0.1% TCA solution on ice. The homogenate was centrifuged at 12,000 rpm for 15 min at 4 °C, and the supernatant was collected for analysis. The reaction mixture (3 mL) contained 1 mL of the extract, 1 mL of 10 mmol/L KIO_4_ solution, and 1 mL of 1 mol/L KI solution. After mixing, the absorbance was measured at 390 nm. The H_2_O_2_ content was calculated using a standard curve and expressed as μmol/g fresh weight (FW) [36].

### 2.6. Antioxidant Activity Measurements

For each treatment, four replicates were used for root measurements. The activities of CAT, SOD, and POD were determined using spectrophotometric methods.

CAT activity: A 0.1 g of fresh root sample was homogenized in 1 mL of 50 mmol/L phosphate buffer (pH 7.8) on ice. The homogenate was centrifuged at 12,000 rpm for 20 min at 4 °C, and the supernatant was collected as the enzyme extract. The reaction mixture (3 mL) contained 2.9 mL of 15 mmol/L H_2_O_2_ solution and 0.1 mL of enzyme extract. The decrease in absorbance at 240 nm was recorded [37].

SOD activity: A 0.1 g of fresh root sample was homogenized in 1 mL of 50 mmol/L phosphate buffer (pH 7.8) on ice. The homogenate was centrifuged at 12,000 rpm for 20 min at 4 °C, and the supernatant was collected as the enzyme extract. The reaction mixture (3 mL) contained 2.7 mL of reaction solution (50 mmol/L phosphate buffer, 130 mmol/L nitro blue tetrazolium, 0.1 mmol/L Na_2_EDTA, and 13 mmol/L methionine). After adding 0.3 mL of enzyme extract, the mixture was exposed to light for 10 min, and the absorbance was measured at 560 nm [37].

POD activity: A 0.1 g of fresh root sample was homogenized in 1 mL of 50 mmol/L phosphate buffer (pH 7.0) on ice. The homogenate was centrifuged at 12,000 rpm for 20 min at 4 °C, and the supernatant was collected as the enzyme extract. The reaction mixture (3 mL) contained 1.9 mL of 50 mmol/L phosphate buffer, 1 mL of 2% guaiacol solution, 0.05 mL of 0.75% H_2_O_2_ solution, and 0.05 mL of enzyme extract. The increase in absorbance at 470 nm was recorded [37].

### 2.7. Data Analysis

Data were processed and analyzed using Excel 2020 and SPSS 22.0 software. One-way analysis of variance (ANOVA) was performed, and Duncan’s multiple range test was used to analyze differences in the indicators of *A. sinicus* under different treatments. GraphPad Prism 10.1.2 was used for plotting.

## 3. Results

### 3.1. Effects of Different Treatments on Root Growth of A. sinicus

**Figure 1:**
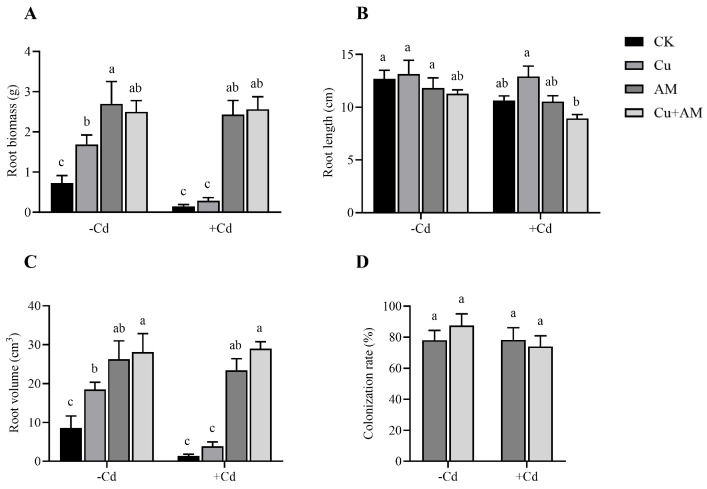
Changes in dry weight (**A**), root length (**B**), root volume (**C**), and mycorrhizal colonization rate (**D**) of *A. sinicus* roots under different treatments. The different letters above the bars, following the data (mean ± standard error), indicate significant differences between treatments (*p* < 0.05). CK, control group; AM, AMF inoculation; Cu, Cu addition; Cu + AM, AMF inoculation and Cu addition; −Cd, without Cd addition in soils; +Cd, with Cd addition in soils.

The roots of *A. sinicus* grew over the 90-day experimental period under all treatments, but the extent of root growth varied significantly among the Cd-stressed, AM fungi inoculated, and exogenous Cu application groups. Cd stress significantly inhibited root growth, while both AM fungi inoculation and exogenous Cu application promoted root development (Figure 1).

Under non-Cd stress conditions, the control group (CK) exhibited the lowest root biomass and root volume. Exogenous Cu application significantly increased these parameters by 131.96% and 114.50%, respectively. Similarly, AM fungi inoculation and the combined Cu + AM treatment also significantly enhanced both parameters, although no significant changes were observed in root length.

Under Cd stress, root parameters all showed obvious declines in *A. sinicus*; compared with the control group, the root biomass, root length, and root volume decreased by 79.38%, 15.98%, and 84.06%, respectively. However, AM fungi inoculation resulted in a dramatic increase in root biomass and root volume by 1521.67% and 1600%, respectively, while root length remained unaffected. Exogenous Cu application increased root length by 21.12% compared to the Cd-stressed control but had no significant impact on biomass or volume. The combined Cu + AM treatment demonstrated the most significant enhancement in these parameters under Cd stress, although it did not significantly affect root length.

### 3.2. Effects of Different Treatments on Cd Concentration, Translocation, and Bioconcentration in the Roots of A. sinicus

**Figure 2:**
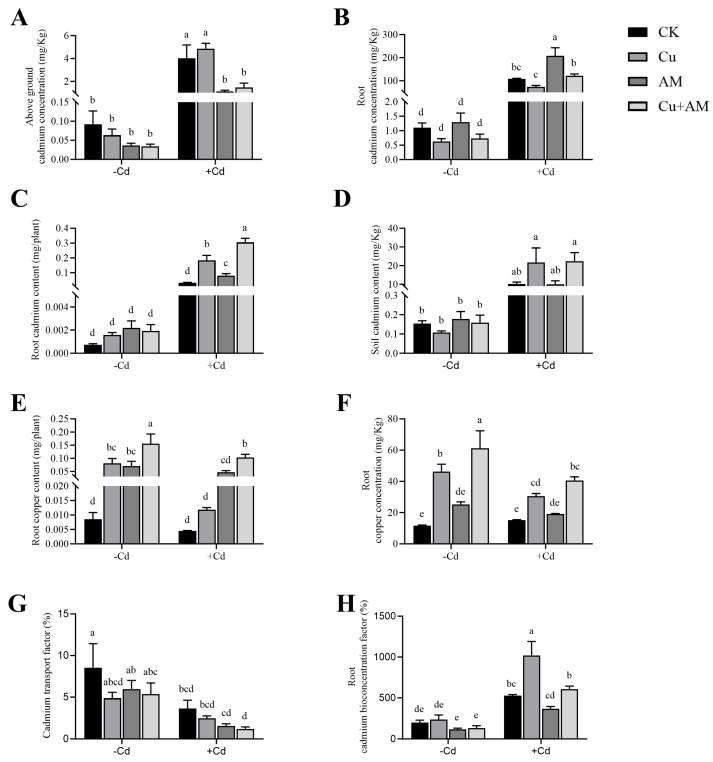
Changes in Cd concentration in the shoots (**A**), Cd concentration in the roots (**B**), Cd content in the shoots (**C**), soil Cd content (**D**), Cu content in the roots (**E**), Cu concentration in the roots (**F**), total Cd translocation factor (**G**), and root Cd bioconcentration factor (**H**) under different treatments. The different letters above the bars, following the data (mean ± standard error), indicate significant differences between treatments (*p* < 0.05). CK, control group; AM, AMF inoculation; Cu, Cu addition; Cu + AM, AMF inoculation and Cu addition; −Cd, without Cd addition in soils; +Cd, with Cd addition in soils.

Under normal (non-Cd) conditions, there were no significant differences in the Cd concentration in the shoots, roots, or soil, nor in the root Cd content among the four treatment groups of *A. sinicus* (Figure 2). However, when AM fungi inoculation and exogenous Cu were applied, both treatments significantly reduced the movement of Cd from the roots to the shoots and promoted Cd accumulation in the roots.

Under Cd stress, AM fungi inoculation led to a 72.4% reduction in the Cd concentration in the shoots, while the combined AM fungi inoculation and Cu application treatment reduced the shoot Cd concentration by 63.32%. Cd stress also decreased the movement of Cd from the roots to the shoots, favoring the accumulation of Cd in the roots. The Cd translocation factor in the leaves decreased significantly, by 57.36% under Cd stress, by 57.70% with AM fungi inoculation, and by 32.96% with Cu application. These findings suggest that both Cu application and AM fungi inoculation were effective in limiting the movement of Cd from the roots to the shoots, with AM fungi inoculation proving more effective than Cu application alone. The combined Cu + AM treatment showed the greatest effect.

When exposed to Cd stress, the root Cd content increased across all treatments. Compared to the control group (CK), AM fungi inoculation boosted root Cd content by 82.26%, Cu application raised it by 59.7%, and the combined Cu + AM treatment led to a remarkable increase of 839.28%. Soil Cd content also significantly increased under Cd stress, rising by 6505.02% compared to the CK group with no added Cd. Under non-Cd conditions, the combined Cu + AM treatment resulted in higher soil Cd content than the AM fungi only treatment. Under Cd stress, Cu application did not significantly change soil Cd content, while AM fungi inoculation significantly increased soil Cd content by 1135.13%. The Cu + AM combination exhibited a synergistic effect, boosting soil Cd content by 1207.39%.

Under non-Cd stress conditions, the application of Cu significantly increased the root Cu content by 850.26% and the concentration by 296.28% (*p* < 0.05). When AM fungi were inoculated, the values were slightly lower compared to the Cu-only group, but the root Cu content was significantly increased by 718.33% (*p* < 0.05) and the concentration by 116.06% compared to the CK group. In the non-stress treatment, the values were the highest in the Cu + AM group, and the root Cu content and concentration showed the order: Cu + AM > Cu > AM. Under Cd stress conditions, Cu + AM significantly increased the root Cu content and concentration by 2170.4% and 167.97%, respectively (*p* < 0.05), but the values were lower compared to those under non-Cd stress conditions.

These results indicate that for Cd accumulation in the roots of *A. sinicus* under Cd stress, the treatments were most effective in the following order: Cu > AM > Cu + AM. The BCF of Cd in the roots increased significantly under Cd stress compared to the non-Cd CK group. The Cd BCF in the roots of AM fungi inoculated plants was 366.08%, which decreased by 30.44% compared to CK. The exogenous Cu treatment increased by 48.33% to 1018.59%, and the Cu + AM treatment group increased by 13.09%. The effectiveness of Cd bioconcentration in the roots followed the order: Cu > Cu + AM > AM.

### 3.3. Changes in the Contents of Osmotic Regulatory Substances in the Roots of A. sinicus Under Different Treatments

**Figure 3:**
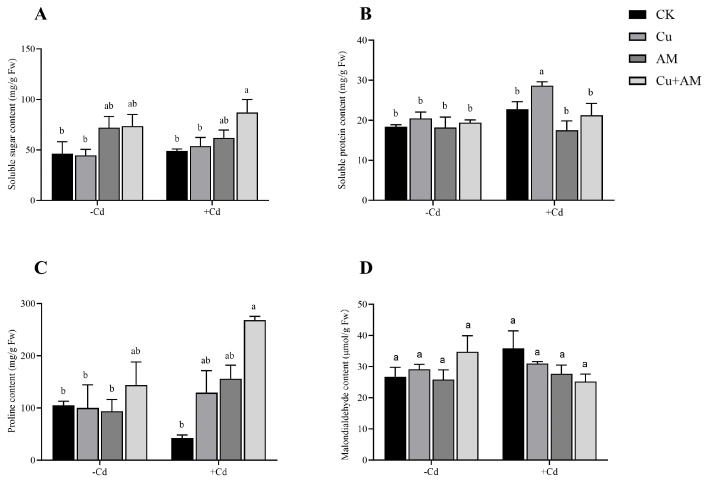
Changes in soluble sugar content (**A**), soluble protein content (**B**), proline content (**C**), and malondialdehyde content (**D**) of the root system of *A. sinicus* under different treatments. The different letters above the bars, following the data (mean ± standard error), indicate significant differences between treatments (*p* < 0.05). CK, control group; AM, AMF inoculation; Cu, Cu addition; Cu + AM, AMF inoculation and Cu addition; −Cd, without Cd addition in soils; +Cd, with Cd addition in soils.

Under non-Cd stress conditions, AM fungi inoculation significantly increased the soluble sugar content in roots by 55.78% compared to the control. Exogenous Cu application showed no significant effects on root osmotic regulatory substances (Figure 3A–C). However, the combined Cu + AM treatment yielded the most pronounced improvement, with soluble sugar content increasing by 59.43%, soluble protein content by 5.7%, and proline content by 36.66%.

Under Cd stress, the contents of soluble sugar and soluble protein in *A. sinicus* roots were elevated. AM fungi inoculation significantly enhanced soluble sugar and proline contents by 26.03% and 268.82%, respectively, while reducing soluble protein content. In contrast, exogenous Cu application increased soluble sugar, soluble protein, and proline contents by 9.36%, 20.68%, and 205.44%, respectively, compared to the Cd-stressed control. The combined Cu + AM treatment exhibited a more pronounced synergistic effect, significantly boosting soluble sugar and proline contents by 77.29% and 445.62%, respectively, though no significant change was observed in soluble protein content.

### 3.4. Changes in Malondialdehyde (MDA) Content in the Roots of A. sinicus Under Different Treatments

Under most treatment conditions, the MDA content in roots showed no significant differences (Figure 3D). In the absence of Cd stress, MDA content in *A. sinicus* roots remained stable under individual AM fungi inoculation or Cu application. However, the combined Cu + AM treatment resulted in a significant increase of 29.87% in MDA content. Under Cd stress, MDA levels were slightly elevated compared to those under non-Cd stress conditions, but AM fungi inoculation, Cu application, and the combined Cu + AM treatment all reduced MDA content.

### 3.5. Changes in H_2_O_2_ Content and Antioxidant Enzyme Activities in the Roots of A. sinicus Under Different Treatments

**Figure 4:**
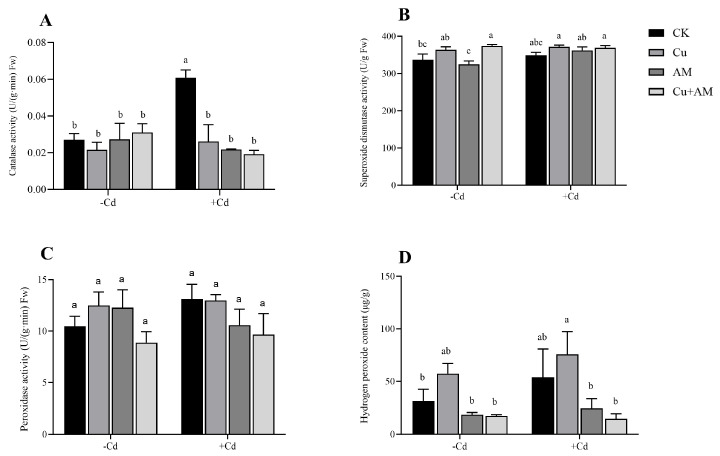
Changes in catalase activity (**A**), superoxide dismutase activity (**B**), peroxidase activity (**C**), and hydrogen peroxide content (**D**) of the root system of *A. sinicus* under different treatments. The different letters above the bars, following the data (mean ± standard error), indicate significant differences between treatments (*p* < 0.05). CK, control group; AM, AMF inoculation; Cu, Cu addition; Cu + AM, AMF inoculation and Cu addition; −Cd, without Cd addition in soils; +Cd, with Cd addition in soils.

Under non-Cd stress conditions, AM fungi inoculation increased POD activity in *A. sinicus* roots by 17.22% and reduced root H_2_O_2_ content by 41.28% compared to the control (Figure 4). The activities of SOD and CAT remained unchanged, showing values similar to those of the control. In the Cu treatment group, SOD activity increased by 8.03%, POD activity by 19.13, and H_2_O_2_ content by 83.52%, whereas CAT activity showed no significant change. The combined Cu + AM treatment had minimal effects, with only slight increases in CAT and SOD activities and decreases in other parameters.

Under Cd stress, the AM fungi and the application of Cu significantly decreased the activity of CAT in *A. sinicus* (*p* < 0.05), and H_2_O_2_ content increased. Specifically, the Cu + AM treatment led to a 74.67% decrease in CAT activity, increased the activity of SOD by 5.81%, while the activities of POD and H_2_O_2_ content decreased by 26.31% and 73.12%, respectively.

## 4. Discussion

Biomass serves as a direct indicator of plant growth and development, with alterations observed under heavy metal stress and AM fungi inoculation [38,39,40]. Previous studies have demonstrated that heavy metals persistently affect plants after soil contamination, whereas AM fungi function as a biological filter to mitigate heavy metal toxicity and enhance plant growth [19]. In *A. sinicus*, Cd stress significantly reduced root biomass, volume, and length. In contrast to Cd, Cu application increased root volume and length without growth inhibition. This phenomenon may be attributed to the non-toxic concentration of Cu applied, as Cu is a vital micronutrient for plants. Earlier research has confirmed that specific Cu concentrations (e.g., 150 mg/kg) can stimulate plant growth [41].

The combined application of AM fungi and Cu significantly increased root biomass, mitigating the negative effects of Cd stress on *A. sinicus* biomass. Moreover, the combined use of AM fungi and Cu enhanced their individual effects on promoting biomass, as supported by the results of Zahangeer et al. (2023) [42] and Saboor et al. (2021) [43]. AM fungi can promote host plants to produce plant growth regulators such as auxin (IAA) and gibberellin (GA) to promote development. *F. mosseae* may secrete specific signaling molecules that trigger the expression of genes related to root development in *A. sinicus*; for example, activate genes involved in IAA biosynthesis and signaling pathways in plant roots, leading to enhanced root growth [44,45,46]. Additionally, the AM fungi plant symbiosis forms arbuscular structures and extraradical hyphae, expanding root absorption capacity and improving nutrient uptake efficiency, thereby increasing biomass [47,48,49]. Consequently, AM fungi inoculation combined with Cu application significantly enhanced both shoot and root biomass of *A. sinicus* under Cd stress.

Plants absorb and accumulate heavy metals from sediments, with the accumulation degree reflected by three indicators: total metal content, BCF, and TF [50,51]. In this study, Cd content in both the aboveground and belowground parts of *A. sinicus* significantly increased under Cd stress in the AM fungi and Cu + AM treatment groups. Generally, higher environmental heavy metal concentrations correlate with greater plant uptake [52]. AM fungi inoculation markedly enhanced Cd accumulation in belowground tissues, likely due to the expansion of AM fungi hyphal network and the secretion of globulin accelerating the binding with Cd; this binding process immobilizes Cd in the rhizosphere, reducing its mobility and availability for plant uptake, while simultaneously facilitating the accumulation of Cd in the root [39,53,54]. Similarly, AM fungi inoculation significantly increased lead absorption in the underground parts of rapeseed and tobacco, doubled copper absorption in reeds, and significantly increased heavy metal content in the roots of Bermuda grass [55,56].

However, Cu and AM fungi application influenced the distribution and translocation of Cd within the plant. Cu application increased Cd content in the aboveground parts but decreased it in the underground parts, possibly due to Cu activating metal ion transport proteins (e.g., the HMA family), which promote Cd translocation to the aboveground parts [57,58]. The results of this study showed that under Cd stress, the TF of *A. sinicus* exceeded 1 in all treatments (TF > 1), indicating its strong bioaccumulation capacity for Cd. Furthermore, the combined application of Cu and AM fungi enhanced Cd bioconcentration in the roots while limiting Cd translocation to the aboveground parts, significantly reducing the TF. This suggests that more Cd was retained in the underground parts. Similarly, the BCF of *A. sinicus* significantly increased under Cd stress, indicating that Cd stress enhanced the plant’s ability to accumulate Cd in the roots. The BCF in the Cu group nearly doubled compared to the control group, possibly because Cu, as an essential micronutrient, activates certain enzymes, such as antioxidant enzymes, enhancing root activity and indirectly promoting Cd absorption. However, AM fungi inoculation reduced the BCF in the roots, possibly because high Cd concentrations inhibited AM fungi activity, weakening their role in promoting Cd absorption.

Osmotic regulation is an adaptive response of plants to stress. Plants increase the concentration of osmotic regulatory substances in cells to maintain normal physiological and metabolic activities. Soluble sugars, soluble proteins, and proline are key osmotic regulatory substances in plants that help maintain osmotic balance, protect cell membrane structures, and alleviate the damage caused by heavy metal stress [59,60]. In this study, the soluble sugar content in the roots of AM fungi treated *A. sinicus* increased significantly under Cd stress, consistent with previous findings [61,62,63]. Similarly, exogenous Cu application increased the contents of soluble sugars, soluble proteins, and proline in the roots of *A. sinicus*, indicating that Cu plays an important role in enhancing the plant’s osmotic regulation capacity. Under Cd stress, Cu may activate the activity of related metabolic enzymes, promoting the synthesis of osmotic regulatory substances (e.g., proline and soluble sugars) while enhancing antioxidant enzyme activity, thereby reducing oxidative damage caused by ROS and protecting the stability of cell membranes and metabolic systems. The increase in soluble protein content due to Cu application may be related to its role in regulating protein synthesis and degradation [64]. The combined application of AM fungi and Cu further enhanced these regulatory effects, stabilizing the osmotic regulation system in the roots of *A. sinicus* and maintaining normal cellular metabolic activities, thereby improving plant tolerance. Studies have shown that AM fungi inoculation effectively increases the osmotic regulatory substances in walnut under salt stress and in *Vitis vinifera* under drought stress, enhancing plant resistance to adverse conditions [65,66]. Plants under stress often experience peroxidation reactions, and MDA, a product of these reactions, can indicate the degree of cell membrane damage and lipid peroxidation [67]. Thus, MDA serves as an important indicator of plant stress resistance [50]. The results of this study showed that Cd stress significantly increased MDA content in the roots of *A. sinicus*, indicating that Cd stress disrupted the normal function of the antioxidant enzyme system in root cells and disturbed the dynamic balance between ROS production and scavenging. Excessive ROS accumulation caused oxidative damage, leading to a significant increase in MDA content and exacerbating cell damage in the roots. Comparing the AM fungi inoculated, Cu-treated, and combined treatment groups, it was found that both AM fungi and Cu reduced MDA content in the roots of *A. sinicus* under Cd stress, with the combined treatment showing the most pronounced effect. This suggests that AM fungi and Cu alleviated cell damage under Cd stress, stabilizing physiological and biochemical processes. *F. mosseae* may secrete antioxidants, like substances that directly scavenge ROS in the root cells of *A. sinicus*. Additionally, it could modulate the expression of genes encoding antioxidant enzymes in the plant, enhancing the plant’s antioxidant capacity and reducing lipid peroxidation, thereby decreasing MDA production [68]. Similarly, studies have shown that AM fungi inoculation significantly improves physiological indicators and reduces MDA content in Sophora davidii and Poplar trees, thereby enhancing host antioxidant capacity [69,70].

Plants possess important protective enzymes, such as CAT, SOD, and POD, which are key components of the plant antioxidant enzyme system [71,72,73,74]. During Cd stress, plants can regulate the activity of these enzymes to mitigate damage. When exposed to Cd stress, plants activate emergency mechanisms: SOD dismutates O_2_^−^ into H_2_O_2_ and O_2_, while POD and CAT catalyze the decomposition of H_2_O_2_ into H_2_O and O_2_. This coordinated interaction reduces ROS accumulation, thereby alleviating heavy metal toxicity under mild stress conditions [75,76]. The results of this study showed that under Cd stress, CAT, SOD, POD activities, and H_2_O_2_ content in the roots of *A. sinicus* increased, indicating that the plant activated its antioxidant system to cope with excessive ROS accumulation induced by Cd. However, after AM fungi inoculation, Cu application, or their combined treatment, CAT and POD activities and H_2_O_2_ content significantly decreased. This may be because these treatments alleviated the intensity of stress, significantly reducing ROS accumulation in the plant and thereby lowering the demand for antioxidant enzymes. AM fungi may alleviate oxidative damage by enhancing plant tolerance to Cd and regulating Cd distribution in the roots, while Cu may directly enhance the function of the antioxidant system to accelerate ROS scavenging. The combined application of AM fungi and Cu exhibited a more pronounced synergistic effect, not only reducing oxidative damage caused by Cd stress but also helping the antioxidant system return to a stable state. Previous studies have found that AM fungi inoculation significantly increased antioxidant enzyme activity in wheat and rice under arsenic stress [77,78].

## 5. Conclusions

**Figure 5:**
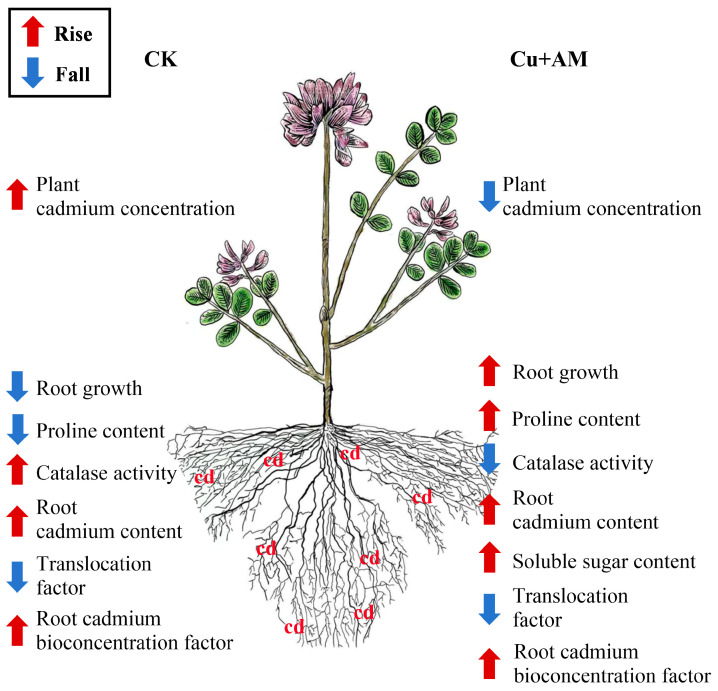
Contrastive analysis of AM fungi inoculation and Cu addition (Cu + AM) and control (CK) in *A. sinicus* response to Cd stress.

This study employed *A. sinicus* as the experimental material for phytoremediation of heavy metal pollution, integrating environmental sustainability and economic feasibility. An *F. mosseae*–*A. sinicus* symbiotic system was established to address limitations in phytoremediation, such as poor plant adaptability and low heavy metal adsorption capacity. The results showed that both Cu application and inoculation with *F. mosseae* significantly promoted the growth of *A. sinicus* under Cd stress and enhanced its Cd tolerance. Notably, the combined *F. mosseae* + Cu treatment exerted a synergistic effect, effectively decreasing the Cd translocation factor and increasing Cd accumulation in roots (Figure 5). These findings indicate that *F. mosseae* interacts with *A. sinicus* to regulate heavy metal uptake and distribution, providing new strategies for phytoremediation of heavy metal-contaminated soils by leveraging the symbiotic relationship between microorganisms and plants.

## Data Availability

The original contributions presented in this study are included in the article. Further inquiries can be directed to the corresponding authors.

## References

[1] Hou D.Y., Jia X.Y., Wang L.W., McGrath S.P., Zhu Y.G., Hu Q., Zhao F.J., Bank M.S., O’Connor D., Nriagu J. (2025). Global soil pollution by toxic metals threatens agriculture and human health. Science.

[2] Tóth G., Hermann T., Szatmári G., Pásztor L. (2016). Maps of heavy metals in the soils of the European Union and proposed priority areas for detailed assessment. Sci. Total Environ..

[3] (2014). The Report on the national general survey of soil contamination. China Environ. Prot. Ind..

[4] Zhang Y., Wang H., Hu M., Cai R., Miao Y., Zhu X. (2024). Heavy metals potentially drive co-selection of antibiotic resistance genes by shifting soil bacterial communities in paddy soils along the middle and lower Yangtze River. Pedosphere.

[5] Chen H.Y., Teng Y.G., Lu S.J., Wang Y.Y., Wang J.S. (2015). Contamination features and health risk of soil heavy metals in China. Sci. Total Environ..

[6] Xu Z., Zhang Q.R., Li X.C., Huang X.F. (2022). A critical review on chemical analysis of heavy metal complexes in water/wastewater and the mechanism of treatment methods. Chem. Eng. J..

[7] You Y.Q., Wang L., Ju C., Wang G., Ma F., Wang Y.J., Yang D.G. (2021). Effects of arbuscular mycorrhizal fungi on the growth and toxic element uptake of *Phragmites australis* (Cav.) Trin. ex Steud under zinc/cadmium stress. Ecotoxicol. Environ. Saf..

[8] Osonubi O. (1994). Comparative effects of vesicular-arbuscular mycorrhizal inoculation and phosphorus fertilization on growth and phosphorus uptake of maize (*Zea mays* L.) and sorghum (*Sorghum bicolor* L.) plants under drought-stressed conditions. Biol. Fertil. Soils.

[9] Bahadur A., Batool A., Nasir F., Jiang S., Mingsen Q., Zhang Q., Pan J., Liu Y., Feng H. (2019). Mechanistic insights into arbuscular mycorrhizal fungi-mediated drought stress tolerance in plants. Int. J. Mol. Sci..

[10] Ouallal I., Abbas Y., ElYacoubi H., Imtara H., Al Zain M.N., Ouajdi M., El Goumi Y., Alzamel N.M., Mohammed Noman O., Rochdi A. (2022). Effects of arbuscular mycorrhizal inoculation by indigenous fungal complexes on the morpho-physiological behavior of Argania spinosa subjected to water deficit stress. Horticulturae.

[11] Pan J., Huang C.H., Luo J., Peng F., Xue X. (2018). Effects of salt stress on plant and the mechanism of arbuscular mycorrhizal fungi enhancing salt tolerance of plants. Adv. Earth Sci..

[12] Yang Y.R. (2015). The Mechanisms by Which Arbuscular Mycorrhizal Fungi (AMF) Enhance Phytoremediation of Soil Heavy Metal Pb Contamination.

[13] Kuang Q.Q., Wu Y.J., Gao Y.M., An T.T., Liu S., Liang L.Y., Xu B.C., Zhang S.Q., Yu M., Shabala S. (2025). Arbuscular mycorrhizal fungi mitigate cadmium stress in maize. Ecotoxicol. Environ. Saf..

[14] Ef A., Abeer H., Alqarawi A., Hend A.A. (2015). Alleviation of adverse impact of cadmium stress in sunflower (*Helianthus annuus* L.) by arbuscular mycorrhizal fungi. Pak. J. Bot..

[15] Vilela L.A.F., Barbosa M.V. (2019). Contribution of Arbuscular Mycorrhizal Fungi in Promoting Cadmium Tolerance in Plants, Cadmium Tolerance in Plants.

[16] Hashem A., Abd_Allah E., Alqarawi A., Al Huqail A.A., Egamberdieva D., Wirth S. (2016). Alleviation of cadmium stress in *Solanum lycopersicum* L. by arbuscular mycorrhizal fungi via induction of acquired systemic tolerance. Saudi J. Biol. Sci..

[17] Wang H.R., Du X.R., Zhang Z.Y., Feng F.J., Zhang J.M. (2023). Rhizosphere interface microbiome reassembly by arbuscular mycorrhizal fungi weakens cadmium migration dynamics. Imeta.

[18] Riaz M., Kamran M., Fang Y.Z., Wang Q.Q., Cao H.Y., Yang G.L., Deng L.L., Wang Y.J., Zhou Y.Y., Anastopoulos I. (2021). Arbuscular mycorrhizal fungi-induced mitigation of heavy metal phytotoxicity in metal contaminated soils: A critical review. J. Hazard. Mater..

[19] Zhao S.P., Yan L., Kamran M., Liu S.S., Riaz M. (2024). Arbuscular mycorrhizal fungi-assisted phytoremediation: A promising strategy for cadmium-contaminated soils. Plants.

[20] Dhalaria R., Kumar D., Kumar H., Nepovimova E., Kuča K., Torequl Islam M., Verma R. (2020). Arbuscular mycorrhizal fungi as potential agents in ameliorating heavy metal stress in plants. Agronomy.

[21] Han Y., Zveushe O.K., Dong F.Q., Ling Q., Chen Y., Sajid S., Zhou L., de Dios V.R. (2021). Unraveling the effects of arbuscular mycorrhizal fungi on cadmium uptake and detoxification mechanisms in perennial ryegrass (*Lolium perenne*). Sci. Total Environ..

[22] Johnson N.C., Wolf J., Koch G.W. (2003). Interactions among mycorrhizae, atmospheric CO_2_ and soil N impact plant community composition. Ecol. Lett..

[23] Xu E.D., Liu Y.Y., Gu D.F., Zhan X.C., Li J.Y., Zhou K.N., Zhang P.J., Zou Y. (2024). Molecular mechanisms of plant responses to copper: From deficiency to excess. Int. J. Mol. Sci..

[24] Migocka M., Malas K., Hossain M.A., Kamiya T., Burritt D.J., Tran L.P., Fujiwara T. (2018). Plant responses to copper: Molecular and regulatory mechanisms of copper uptake, distribution and accumulation in plants. Plant Micronutrient Use Efficiency: Molecular and Genomic Perspectives in Crop Plants.

[25] Gong Q., Li Z.H., Wang L., Zhou J.Y., Kang Q., Niu D.D. (2021). Gibberellic acid application on biomass, oxidative stress response, and photosynthesis in spinach (*Spinacia oleracea* L.) seedlings under copper stress. Environ. Sci. Pollut. Res..

[26] Adrees M., Ali S., Rizwan M., Ibrahim M., Abbas F., Farid M., Zia-ur-Rehman M., Irshad M.K., Bharwana S.A. (2015). The effect of excess copper on growth and physiology of important food crops: A review. Environ. Sci. Pollut. Res..

[27] Ruscitti M., Arango M., Beltrano J. (2017). Improvement of copper stress tolerance in pepper plants (*Capsicum annuum* L.) by inoculation with arbuscular mycorrhizal fungi. Theor. Exp. Plant Physiol..

[28] Wang S.Z., Jin Z.X., Li Y.L., Gu Y.F. (2015). Effects of arbuscular mycorrhizal fungi inoculation on the photosynthetic pigment contents, anti-oxidation capacity and membrane lipid peroxidation of *Elsholtzia splendens* leaves under copper stress. Acta Ecol. Sin..

[29] Pérez R., Tapia Y., Antilén M., Ruiz A., Pimentel P., Santander C., Aponte H., González F., Cornejo P. (2023). Beneficial interactive effects provided by an arbuscular mycorrhizal fungi and yeast on the growth of *Oenothera picensis* established on Cu mine tailings. Plants.

[30] Chen X.H., Zhao B. (2007). Arbuscular mycorrhizal fungi mediated uptake of lanthanum in Chinese milk vetch (*Astragalus sinicus* L.). Chemosphere.

[31] Li Y., Peng J., Shi P., Zhao B. (2009). The effect of Cd on mycorrhizal development and enzyme activity of Glomus mosseae and Glomus intraradices in *Astragalus sinicus* L.. Chemosphere.

[32] Peng J., Li Y., Shi P., Chen X.H., Lin H., Zhao B. (2011). The differential behavior of arbuscular mycorrhizal fungi in interaction with *Astragalus sinicus* L. under salt stress. Mycorrhiza.

[33] Řezáčová V., Némethová E., Stehlíková I., Czakó A., Gryndler M. (2023). Arbuscular mycorrhizal fungus *Funneliformis mosseae* improves soybean growth even in soils with good nutrition. Microbiol. Res..

[34] Xu Z., Wu Y., Xiao Z., Ban Y., Belvett N. (2019). Positive effects of *Funneliformis mosseae* inoculation on reed seedlings under water and TiO_2_ nanoparticles stresses. World J. Microbiol. Biotechnol..

[35] Lu R.R., Hu Z.H., Zhang Q.L., Li Y.Q., Lin M., Wang X.L., Wu X.N., Yang J.T., Zhang L.Q., Jing Y.X. (2020). The effect of *Funneliformis mosseae* on the plant growth, Cd translocation and accumulation in the new Cd-hyperaccumulator *Sphagneticola calendulacea*. Ecotoxicol. Environ. Saf..

[36] Zhu X., Song F., Xu H. (2010). Influence of arbuscular mycorrhiza on lipid peroxidation and antioxidant enzyme activity of maize plants under temperature stress. Mycorrhiza.

[37] Zhou W., Zhang M., Tao K., Zhu X. (2022). Effects of arbuscular mycorrhizal fungi and plant growth-promoting rhizobacteria on growth and reactive oxygen metabolism of tomato fruits under low saline conditions. Biocell.

[38] Miransari M. (2011). Hyperaccumulators, arbuscular mycorrhizal fungi and stress of heavy metals. Biotechnol. Adv..

[39] Pogrzeba M. (2024). Inoculation with Arbuscular mycorrhizal fungi supports the uptake of macronutrients and promotes the growth of *Festuca ovina* L. and *Trifolium medium* L., a candidate species for green urban infrastructure. Plants.

[40] Garg N., Singh S., Kashyap L., Varma A., Prasad R., Tuteja N. (2017). Arbuscular mycorrhizal fungi and heavy metal tolerance in plants: An insight into physiological and molecular mechanisms. Mycorrhiza-Nutrient Uptake, Biocontrol, Ecorestoration.

[41] Liu S.J., Ouyang X.L., Yang A.H., Liu T.Y., Liu L.P., Zhou H. (2024). Growth and physiological response of *Cinnamomum camphora* (L.) Presl to copper stress and analysis of copper enrichment and transport characteristics. Plant Sci. J..

[42] Zahangeer A.M., Rabia C.T., Mridha M.A.U. (2023). Arbuscular mycorrhizal fungi enhance biomass growth, mineral content, and antioxidant activity in tomato plants under drought stress. J. Food Qual..

[43] Saboor A., Ali M.A., Hussain S., El Enshasy H.A., Hussain S., Ahmed N., Gafur A., Sayyed R., Fahad S., Danish S. (2021). Zinc nutrition and arbuscular mycorrhizal symbiosis effects on maize (*Zea mays* L.) growth and productivity. Saudi J. Biol. Sci..

[44] Khalloufi M., Martínez-Andújar C., Lachaâl M., Karray-Bouraoui N., Pérez-Alfocea F., Albacete A. (2017). The interaction between foliar GA3 application and arbuscular mycorrhizal fungi inoculation improves growth in salinized tomato (*Solanum lycopersicum* L.) plants by modifying the hormonal balance. J. Plant Physiol..

[45] Liu R.C., Yang L., Zou Y.N., Wu Q.S. (2023). Root-associated endophytic fungi modulate endogenous auxin and cytokinin levels to improve plant biomass and root morphology of trifoliate orange. Hortic. Plant J..

[46] Liu C.Y., Zhang F., Zhang D.J., Srivastava A., Wu Q.S., Zou Y.N. (2018). Mycorrhiza stimulates root-hair growth and IAA synthesis and transport in trifoliate orange under drought stress. Sci. Rep..

[47] Bücking H., Kafle A. (2015). Role of arbuscular mycorrhizal fungi in the nitrogen uptake of plants: Current knowledge and research gaps. Agronomy.

[48] Qiao X., Bei S.K., Li C.J., Dong Y., Li H.G., Christie P., Zhang F., Zhang J.L. (2015). Enhancement of faba bean competitive ability by arbuscular mycorrhizal fungi is highly correlated with dynamic nutrient acquisition by competing wheat. Sci. Rep..

[49] Zhang L., Shi N., Fan J.Q., Wang F., George T.S., Feng G. (2018). Arbuscular mycorrhizal fungi stimulate organic phosphate mobilization associated with changing bacterial community structure under field conditions. Environ. Microbiol..

[50] Du J.Q., Zhang B.G., Li J.X., Lai B. (2020). Decontamination of heavy metal complexes by advanced oxidation processes: A review. Chin. Chem. Lett..

[51] Chi K.Y., Zou R., Wang L., Huo W.M., Fan H.L. (2019). Cellular distribution of cadmium in two amaranth (*Amaranthus mangostanus* L.) cultivars differing in cadmium accumulation. Environ. Sci. Pollut. Res..

[52] Wang J., Hu Y.F. (2023). Translocation and accumulation of heavy metals from the rhizoshphere soil to the medicinal plant (*Paeonia Lactiflora* Pall.) grown in Bozhou, Anhui Province, China. Environ. Pollut. Bioavailab..

[53] Khanna K., Kohli S.K., Ohri P., Bhardwaj R., Ahmad P. (2022). Agroecotoxicological aspect of Cd in soil–plant system: Uptake, translocation and amelioration strategies. Environ. Sci. Pollut. Res..

[54] Rask K.A., Johansen J.L., Kjøller R., Ekelund F. (2019). Differences in arbuscular mycorrhizal colonisation influence cadmium uptake in plants. Environ. Exp. Bot..

[55] Wang F.Y., Wang L., Shi Z.Y., Li Y.J., Song Z.M. (2012). Effects of AM inoculation and organic amendment, alone or in combination, on growth, P nutrition, and heavy-metal uptake of tobacco in Pb-Cd-contaminated soil. J. Plant Growth Regul..

[56] Zhan F.D., Li B., Jiang M., Li T.G., He Y.M., Li Y., Wang Y.S. (2019). Effects of arbuscular mycorrhizal fungi on the growth and heavy metal accumulation of bermudagrass [*Cynodon dactylon* (L.) Pers.] grown in a lead–zinc mine wasteland. Int. J. Phytoremediation.

[57] Cao Y.Q., Nie Q.K., Gao Y., Xu Z.C., Huang W.X. (2018). The studies on cadmium and its chelate related transporters in plants. Crops.

[58] Yruela I. (2009). Copper in plants: Acquisition, transport and interactions. Funct. Plant Biol..

[59] Lenoir I., Fontaine J., Sahraoui A.L.H. (2016). Arbuscular mycorrhizal fungal responses to abiotic stresses: A review. Phytochemistry.

[60] Khan N., Ali S., Zandi P., Mehmood A., Ullah S., Ikram M., Ismail I., Babar M. (2020). Role of sugars, amino acids and organic acids in improving plant abiotic stress tolerance. Pak. J. Bot..

[61] Yooyongwech S., Samphumphuang T., Tisarum R., Theerawitaya C., Cha-Um S. (2016). Arbuscular mycorrhizal fungi (AMF) improved water deficit tolerance in two different sweet potato genotypes involves osmotic adjustments via soluble sugar and free proline. Sci. Hortic..

[62] Wu B.H., Zeng Z.F., Wu X.Y., Li Y.Y., Wang F.Q., Yang J., Li X. (2022). Jasmonic acid negatively regulation of root growth in Japonica rice (*Oryza sativa* L.) under cadmium treatment. Plant Growth Regul..

[63] Afzal S., Chaudhary N., Singh N.K., Aftab T., Hakeem K.R. (2021). Role of soluble sugars in metabolism and sensing under abiotic stress. Plant Growth Regulators: Signalling Under Stress Conditions.

[64] Cui X.M., Wu X.B., Li X.Y., Li X.H. (2011). Responses of growth, functional enzyme activity in biomembrane of tomato seedlings to excessive copper, cadmium and the alleviating effect of exogenous nitric oxide. J. Plant Nutr. Fertil..

[65] Zhou Y.H., Wei M., Li Y.P., Tang M., Zhang H.Q. (2023). Arbuscular mycorrhizal fungi improve growth and tolerance of *Platycladus orientalis* under lead stress. Int. J. Phytoremediation.

[66] Ye Q.H., Wang H., Li H. (2023). Arbuscular mycorrhizal fungi enhance drought stress tolerance by regulating osmotic balance, the antioxidant system, and the expression of drought-responsive genes in *vitis vinifera* L.. Aust. J. Grape Wine Res..

[67] Hasanuzzaman M., Hossain M.A., da Silva J.A.T., Fujita M., Bandi V., Shanker A.K., Shanker C., Mandapaka M. (2012). Plant response and tolerance to abiotic oxidative stress: Antioxidant defense is a key factor. Crop Stress and Its Management: Perspectives and Strategies.

[68] Liang S.M., Hashem A., Abd-Allah E.F., Wu Q.S. (2023). Root-associated symbiotic fungi enhance waterlogging tolerance of peach seedlings by increasing flavonoids and activities and gene expression of antioxidant enzymes. Chem. Biol. Technol. Agric..

[69] Zhao L.L., Wang L.T., Chen K.K., Sun H., Wang P.C. (2024). Effects of arbuscular mycorrhizal fungi on the growth and physiological performance of *Sophora davidii* seedling under low-phosphorus stress. J. Plant Growth Regul..

[70] Xu N., Wei X.W., Wang Y., Dong J.X., Yang X.C. (2025). Mechanism of arbuscular mycorrhizal fungi in enhancing lead stress resistance in poplar trees. Forests.

[71] Bhaduri A.M., Fulekar M. (2012). Antioxidant enzyme responses of plants to heavy metal stress. Rev. Environ. Sci. Biotechnol..

[72] Sheng L., Sun X., Mo C., Hao M., Wei X., Ma A. (2023). Relationship between antioxidant enzymes and sclerotial formation of Pleurotus tuber-regium under abiotic stress. Appl. Microbiol. Biotechnol..

[73] Kapoor D., Singh S., Kumar V., Romero R., Prasad R., Singh J. (2019). Antioxidant enzymes regulation in plants in reference to reactive oxygen species (ROS) and reactive nitrogen species (RNS). Plant Gene.

[74] Anass K., Zoulfa R., Azzouz K., Nada N., Abdelhamid E., Bouchra B., Ayoub K., Mohammed E.M., Naima N., Nhiri M. (2024). Effects of mycorrhizal symbiosis and Ulva lactuca seaweed extract on growth, carbon/nitrogen metabolism, and antioxidant response in cadmium-stressed sorghum plant. Physiol. Mol. Biol. Plants.

[75] Li W.T., Chen K., Li Q., Tang Y.L., Jiang Y.Y., Su Y. (2023). Effects of arbuscular mycorrhizal fungi on alleviating cadmium stress in *Medicago truncatula* Gaertn. Plants.

[76] Yan L., Du C.Q., Riaz M., Jiang C.C. (2019). Boron mitigates citrus root injuries by regulating intracellular pH and reactive oxygen species to resist H^+^-toxicity. Environ. Pollut..

[77] Mitra D., Saritha B., Janeeshma E., Gusain P., Khoshru B., Nouh F.A.A., Rani A., Olatunbosun A.N., Ruparelia J., Rabari A. (2022). Arbuscular mycorrhizal fungal association boosted the arsenic resistance in crops with special responsiveness to rice plant. Environ. Exp. Bot..

[78] Sharma S., Anand G., Singh N., Kapoor R. (2017). Arbuscular mycorrhiza augments arsenic tolerance in wheat (*Triticum aestivum* L.) by strengthening antioxidant defense system and thiol metabolism. Front. Plant Sci..

